# Variations of the Elastic Properties of the CoCrFeMnNi High Entropy Alloy Deformed by Groove Cold Rolling

**DOI:** 10.3390/ma11081337

**Published:** 2018-08-02

**Authors:** Paul Lohmuller, Laurent Peltier, Alain Hazotte, Julien Zollinger, Pascal Laheurte, Eric Fleury

**Affiliations:** 1 Laboratoire d’Etude des Microstructures et de Mécanique des Matériaux, UMR 7239 CNRS, Arts et Métiers ParisTech Campus de Metz, Université de Lorraine, 7 rue Félix Savart, F-57073 Metz, CEDEX 03, France; paul.lohmuller@univ-lorraine.fr (P.L.); laurent.peltier@ensam.eu (L.P.); alain.hazotte@univ-lorraine.fr (A.H.); pascal.laheurte@univ-lorraine.fr (P.L.); 2 Institut Jean Lamour, UMR 7198 CNRS, Université de Lorraine, Campus ARTEM, F-54011 Nancy, CEDEX 01, France; julien.zollinger@univ-lorraine.fr; 3 LABoratory of EXcellence Design of Alloy Metals for low-mAss Structures, Université de Lorraine, F-54011 Nancy, CEDEX 01, France

**Keywords:** high entropy alloy, crystallographic texture, groove rolling, elastic properties

## Abstract

The variations of the mechanical properties of the CoCrFeMnNi high entropy alloy (HEA) during groove cold rolling process were investigated with the aim of understanding their correlation relationships with the crystallographic texture. Our study revealed divergences in the variations of the microhardness and yield strength measured from samples deformed by groove cold rolling and conventional cold rolling processes. The crystallographic texture analyzed by electron back scattered diffraction (EBSD) revealed a hybrid texture between those obtained by conventional rolling and drawing processes. Though the groove cold rolling process induced a marked strengthening effect in the CoCrFeMnNi HEA, the mechanical properties were also characterized by an unusual decrease of the Young’s modulus as the applied groove cold rolled deformation increased up to about 0.5 before reaching a stabilized value. This decrease of the Young’s modulus was attributed to the increased density of mobile dislocations induced by work hardening during groove cold rolling processing.

## 1. Introduction

High entropy alloys (HEAs), a new class of metallic materials in which the stability of the solid solution is explained by contribution of the configurational entropy, have recently received particular attention, owing to the prospect of developing new systems with tailored properties particularly suitable for a large range of different applications from dies and molding materials to corrosion-resistant coatings in manufacturing, energy, transport, and aeronautical industries [1,2,3,4]. The stabilization of the solid solution is also associated to special features such as low diffusion kinematics, lattice distortion and “cocktail effect” whose effects have been discussed in the literature [1,3,5].

The equi-atomic CoCrFeMnNi composition developed by Cantor and colleagues [6] is currently one of the most studied HEA. Though its mechanical properties are not outstanding [7,8,9], this alloy characterized by a single solid solution of face centered cubic structure has appeared attractive as a model material to gain a better understanding on characteristics particular to solid solution HEA alloys against those of conventional solid solution alloys. Some key studies, such as those published by Wu et al. [10] and Laurent-Brocq and co-workers [11,12], already helped in the understanding the formation of these solid solutions, particularly the influence of each constituent element on the stability of the microstructure. The mechanical properties of HEAs, as those of conventional metallic materials, are also controlled by the microstructure. If several studies have revealed the dependency of the mechanical properties with the grain size [7,13,14], a few have recently reported the variation of the crystallographic texture during mechanical processing of HEAs. For example, conventional cold rolling [15,16,17,18], rotary swaging [19], or severe plastic deformation [20] have been investigated and the results of these studies show characteristics very similar to those obtained with low stacking fault energy (SFE) materials such as brass alloys [21,22]. Examinations of the deformation mechanisms have demonstrated that deformation occurred mainly by dislocation glide and sub-cell creation [8,18]. However, under particular conditions, such as tests performed under high strain level or at cryogenic temperature [16,18,23,24], the CoCrFeMnNi also presented deformation twinning, which can be seen as a secondary deformation mechanism.

Though most of studies are concerned about the mechanical properties, the modifications of the Young’s modulus induced by work hardening have not been intensively studied. However, as seen earlier in the 1950′s, in their theory of work hardening, both Mott [25] and Friedel [26] took into account a network of dislocations in face-centered cubic crystals to explain the changes in the elastic modulus upon work hardening. More recently, others authors have proposed to account for the increase of internal stresses during deformation, the development of crystallographic texture and change in the dislocation arrangement [27] to describe the variation of solid solution alloys.

This study was thus undertaken to investigate the evolution of the hardness and tensile properties of CoCrFeMnNi HEA alloy with the increasing area reduction achieved by grooved cold rolling. Though this process resulted in a marked strengthening effect, it was also accompanied by a reduction of the Young’s modulus. The variation of the crystallographic texture with the increasing groove rolling deformation was found to be slightly different from that obtained during conventional cold rolling. This study attempted to provide elements enabling the understanding of the observed changes in the mechanical properties.

## 2. Materials and Methods

### 2.1. Samples Production

A 5 kg ingot of the equi-atomic CoCrFeMnNi alloy of composition Co_20_._1_Cr_19_._9_Fe_20_._6_Mn_19_._5_Ni_19_._9_ (in at.%) as analyzed by electron dispersive spectroscopy (EDS) was produced by vacuum arc melting at KIST, Korea, using initial elements with purity above 99.9%. Hot forging treatment and hot rolling at 1000 °C were performed to homogenize the microstructure and to reduce the initial ingot thickness down to 12 mm. After another thickness reduction by conventional cold rolling, 4 samples of dimension 9.3 × 9.3 × 80 mm^3^ were machined perpendicularly to the rolling direction. Finally an annealing heat treatment was then applied during 3600 s at 900 °C under a protective Argon atmosphere. Following this heat treatment, samples were considered, for this study, in a reference state in term of microstructure and mechanicals properties.

**Figure 1:**
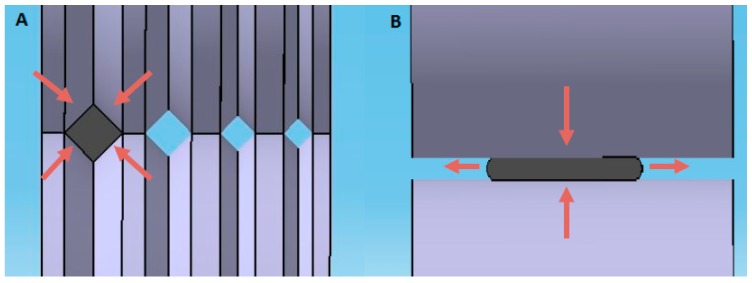
Grooved cold rolling (**A**) versus conventional rolling (**B**), the red arrows correspond to the in plane displacement direction induced by the process.

Room temperature groove cold rolling process was performed on the annealed square bars. In this process, rollers present V-grooves of different dimensions as illustrated in Figure 1A. These grooves result in a bidirectional loading on the square sections of the samples, which prevents transversal displacement. This transversal displacement, though moderate, is inherent to the conventional rolling process (Figure 1B). From this viewpoint, groove cold rolling may be seen as a hybrid process between conventional rolling and swaging or extrusion.

The samples were respectively rolled down to 14% and 33% area reduction ratios, i.e., rolling deformation ε = 0.2 and ε = 0.4, respectively. The obtained square wire-type samples were cut off in order to investigate the tensile properties (minimal length about 90 mm). The rests of the samples were rolled to successively up to 89% area reduction ratio (i.e., rolling deformation ε = 2.2), corresponding to a final section of about 3.1 × 3.1 mm^2^. For each deformation level, a wire was extracted with a minimal length of about 120 mm for tensile test and microhardness measurements. In order to determine the tensile properties of the initial state, the last sample was rolled until 38% area reduction ratio (i.e., rolling deformation ε = 0.7) and annealed under the same conditions as described above. Table 1 summarizes the thickness of each sample, the associated reduction state calculated by $\varphi =100\times \left(1-e_{f}{}^{2}/e_{i}{}^{2}\right)$ and the true deformation obtained by $\;\varepsilon \;=\;2\times \ln(e_{i}/e_{f})$ [28], where *e_i_* and *e_f_* correspond to the initial and final thicknesses, respectively.

### 2.2. Mechanical Testing

**Figure 2:**
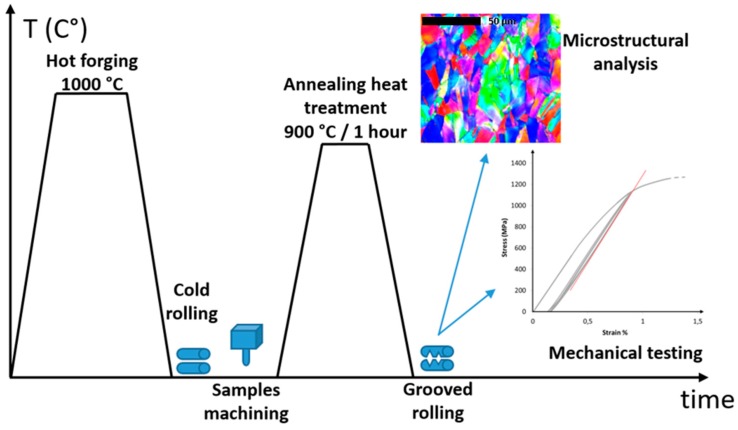
Graphical representation of samples production procedure and characterization.

Uniaxial tensile tests were carried out on 100 kN universal Zwick tensile test machine, using a 50 kN load cell. With the aim of determining the Young’s modulus, samples were first loaded to 1% strain, then 5 charge/discharge cycles were performed. Strains were measured using an Epsylon extensometer with a gage length of 25 mm, and the samples were deformed at a constant strain rate of 1 × 10^−3^ s^−1^. Values of the Young’s modulus were determined as the mean of the initial slope of each discharging portion of the curves. Values of the yield strength were obtained from the conventionally 0.2% of plastic deformation. A typical obtained curve is shown in the right-hand side inset in Figure 2.

For microstructural observations, samples were polished (along the rolling direction) using SiC papers with grit size about 80 in order to reduce the thickness and to reach approximately the center part of the samples. Then surfacing polishing was performed with grit size from 1000 to 4000. Hardness values were measured by micro-indentation Vickers tests carried out using a Zwick Rowel machine with a load of 0.3 gf, leading to indentation size of about 35 to 70 µm. Distance between adjacent measurements was at least 200 µm. For each sample, the hardness value was averaged from 15 measurements, and the errors bars correspond to the measured standard deviation.

### 2.3. Microstructural Analysis

Before tensile testing, pieces of rolled materials were cut off for microstructural analyses. Samples were polished using SiC paper with grit size down to 4000 followed by a Al_2_O_3 _polishing with final particle size of 0.1 µm. Microstructural analyses were carried out on SEM Jeol 6490, at 20 kV with a working distance of 21 mm. Electron back scattered diffraction (EBSD) analyses were performed by Oxford instrument detector with a minimal number of detected Kikuchi bands equal to 8 and a step size of 0.4 µm. EBSD were studied in four cases: after annealing and after section reduction of ε = 0.5, 1.1 and 2.2. EBSD maps, Figure pole (FP), inverse figure pole (IFP) and orientation density function (ODF) were obtained using ATEX software developed at LEM3 [29]. Texture components were also performed using a spread of 15° from their ideal orientation to compute their volume fractions. All the obtained texture components were summed in order to determine the randomly oriented components by evaluating the difference between this sum and the totality [16,18]. The different analyzed samples are indicated in Table 1. The whole procedure from hot forging to EBSD analysis is summarized in Figure 2.

## 3. Results

### 3.1. Microhardness and Tensile Results

**Figure 3:**
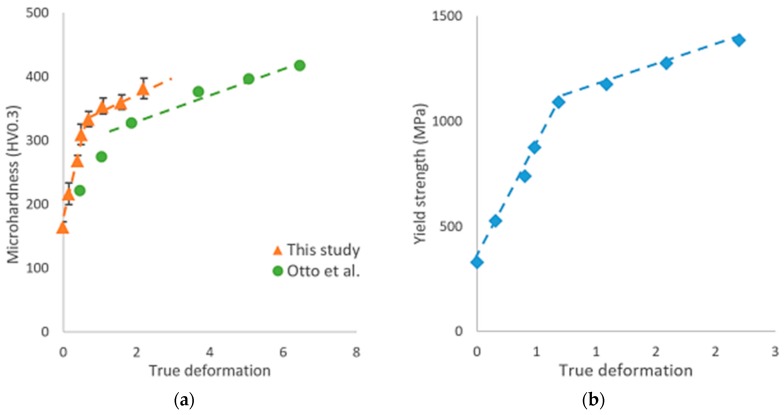
(**a**) Microhardness Vickers (HV_0.3_) evolution with the surface reduction, for this study (orange triangle) and comparison with results published by Otto et al. [15] (green dot), (**b**) yield strength evolution during grooved cold rolling process.

Figure 3a shows the variations of the microhardness values and associated standard deviations for the different deformation states applied by groove cold rolling and data obtained by Otto et al. [15] on CoCrFeMnNi HEA processed by conventional cold rolling have been added for comparison. In the as-annealed state, the value of the microhardness was about 165 HV_0.3_, which is in good agreement with values obtained in published articles under the same annealing treatment [8,23]. The variation of the microhardness can be described by two different linear curves with a breakdown at a level of deformation of about 0.5. From the initial annealing state to a condition corresponding to the groove rolling deformation ε ~ 0.5, the microhardness displayed a marked increased from 165 HV_0.3_ to about 310 HV_0.3_, characterized by a larger value of the slope (284 kgf/mm^2^) than those reported by Otto et al. [8] (92 kgf/mm^2^) represented by the green dashed line in the Figure 3a. For values of the groove rolling deformation beyond 0.5, the microhardness continued to increase however with a reduced slope until reaching a value of 380 HV_0.3_ for a groove rolling deformation ε = 2.2. It is remarkable that the microhardness values followed the same trend under both groove rolled and conventional rolling processes, though the initial slope was larger and that the breakdown in the slope occurred earlier for samples deformed by groove rolled process.

The evolution of the yield strength, as shown in Figure 3b, followed a similar trend to that of the microhardness. Values of the yield strength obtained in the annealed condition are in good agreement with those reported in references [7,8]. The high values of the yield strength obtained after large area reduction were also found to be in accordance with the values reported by Sun et al. for CoCrFeMnNi HEA samples with fine-grained microstructure [7].

**Figure 4:**
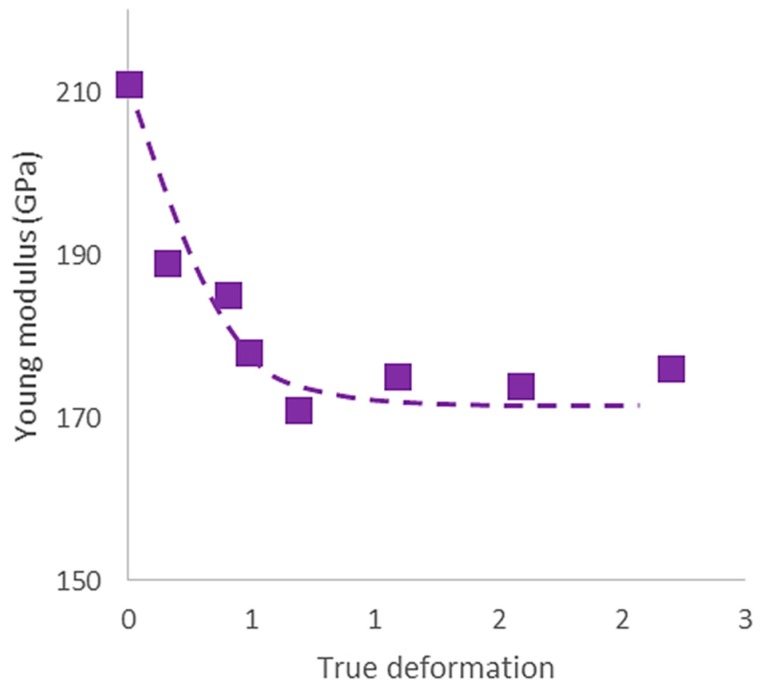
Young’s modulus variation with the thickness reduction during grooved cold rolling (maximal incertitude ± 6 GPa).

As mentioned in the introduction, a part of this study focused on understanding the evolution of the Young’s modulus as function of the groove rolling deformation. Figure 4 indicates that the value obtained in the as-annealed sample, i.e., 210 GPa, is also in agreement with those reported in the literature [9,30]. It also shows that the value decreased down to about 175 GPa, as the groove rolling deformation reached 0.5, which is corresponding to approximately a 15% decrease in the Young’s modulus. As far as we know, no similar results have to be reported on deformed HEA. To the authors’ knowledge, only a few studies have attempted to correlate the variations of the Young’s modulus of engineering materials with the increase of cold plastic deformation [27,31,32]. As mentioned by Benito et al. [27], four possible contributions have been proposed to explain the decrease in Young’s modulus: (i) debonding between a matrix and a second-phase or inclusions [33,34]; (ii) increase in the internal stresses during plastic deformation [35]; (iii) introduction and development of texture during deformation [27,31]; and (iv) change in dislocation arrangement and kinematics [27,31].

By considering the single-phase character of CoCrFeMnNi HEA, the first mentioned reason appeared to be irrelevant. In their study, Benito et al. have suggested that the internal stresses generated during the early stage of cold rolling were not sufficient to describe the variation of the Young’s modulus [27]. Consequently, the decrease in the Young’s modulus during the early stage of groove cold rolling could result from either a crystallographic texture effect or a change in the dislocation arrangement and deformation mechanism.

The different mechanisms in the CoCrFeMnNi HEA are now well documented in the literature for different deformation modes and different degrees of deformation [7,8,18,23,36,37]. From these studies, it appears that the deformation mechanisms are governed by dislocation glide upon low strain value, and by dislocation glide combined with twinning upon high level of plastic deformation. In an attempt to elucidate the decrease of the Young’s Modulus, a thorough study of the evolution of the crystalline texture as a function of the section reduction has thus been undertaken.

### 3.2. Texture Analysis

**Figure 5:**
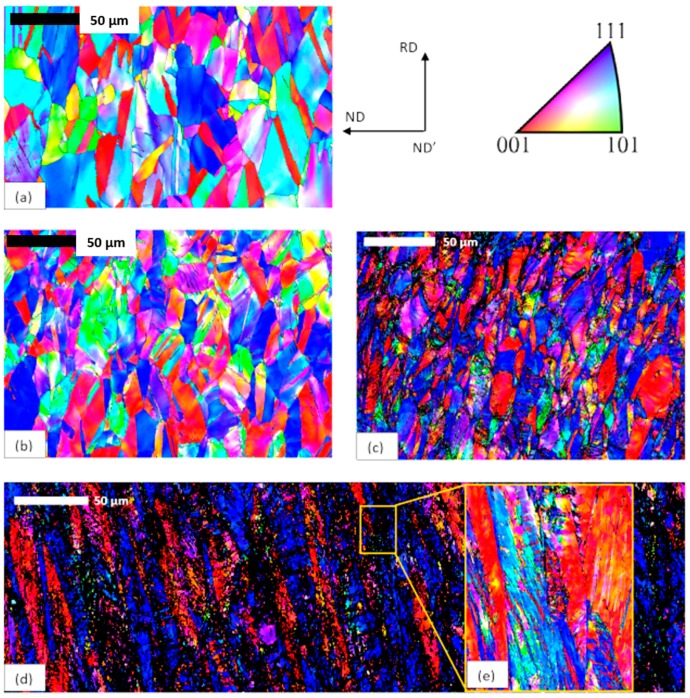
EBSD-band contrast maps described by inverse figure pole (IFP) maps (upper right corner): (**a**) as-annealed; (**b**) after ε = 0.5 by groove cold rolling; (**c**) after ε = 1.1 groove cold rolling; (**d**) ε = 2.2 groove cold rolling; and (**e**) focused area of the image (**d**). Note that magnification is the same for all maps (maps (**d**) corresponding to a stack of 2 EBSD maps with a step size of about 0.2 µm).

Figure 5 shows the EBSD maps obtained for the annealed state and for samples after three different values of the area reduction, i.e., ε = 0.5, 1.1, and 2.2. The initial state microstructure consisted of equiaxed grains with a mean grain diameter about 27 µm (as computed by ATEX). Figure 5b,d show the evolution of the microstructure with increasing strain induced by the groove cold rolling process. It can be observed that grains were progressively elongated along the rolling direction (RD) and compressed in the normal direction (ND). For samples processed under low deformation level (Figure 5b), some deformation features oriented at 45° to the rolling direction appeared and their fraction progressively increased with the increasing level of groove rolling deformation (Figure 5c,d). Figure 5e shows a focused area of the Figure 5d obtained with a step size of 0.05 µm and a high indexation rate. These features resulted from different deformation mechanisms operating in CoCrFeMnNi HEA that are dislocation glide and twinning as already described in different studies [7,8,19,36].

**Figure 6:**
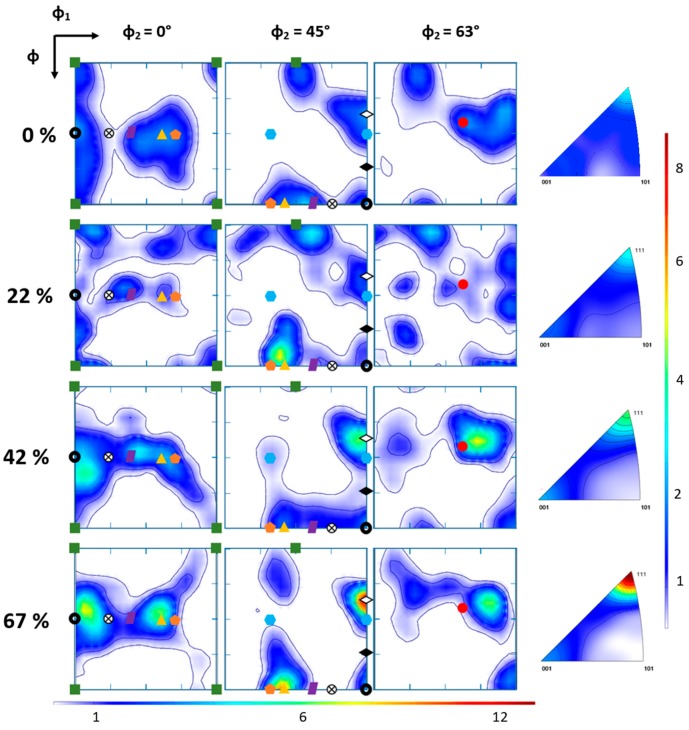
Orientation density function (ODF) section at φ_2_ = 0°, 45° and 63° (right) at each analyzed deformation state and their respective IFP along rolling direction, red arrows indicate maximal intensity for orientation {111} and {001}. Horizontal and vertical bars, respectively, correspond to ODF and IFP maximal intensity. The red dashed line represent the α-fiber. The symbols for each texture component are, respectively, Cb: 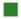, G: 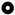, B: 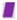, G/B: ⨂, Cu: <>, A: 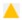, F: 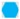, P: 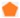, CuT: ◆, S: 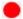, as defined in Table 2.

Figure 6 shows the evolution of the ODF section intensity and IFP intensity along the rolling direction. The map of the sample in the as-annealed state shows a slight recrystallization texture of the cube on face Cb {001} <100> type as well as a partial α-fiber (<110> along ND). Such texture has been reported for CoCrFeMnNi HEA deformed by conventional cold rolling or hot forging prior to an annealing treatment at 900 °C during 1 h [11,18]. One may also note that recrystallized twinning occurred during the annealing treatment as reported in [16,18].

The Figure 6, showing the IFP of the deformed state, indicates a strong increase of the <111> fiber texture along RD as the level of deformation induced by groove cold rolling increased. This fiber texture is associated to the <001> fiber, which is a minor component of the texture, as indicated by local maximal intensity. Both fiber textures are frequently observed for FCC materials [38,39], and have also been reported for CoCrFeMnNi HEA [19] deformed to swaging or drawing deformation. These observations based on IFP may be balanced with the analyses of the ODF sections. In the ODF map, the α-fiber component (<110> along ND, not shown in the IFP (Figure 6) is represented by the red dashed line. The <111> and <001> directions along RD are highlighted by, respectively, the (Copper (Cu), A) and (Cube (Cb), Goss (G)) texture components in the ODF with φ_2_ = 45° (referred in Table 2) [40].

For the sample processed to a groove rolling deformation ε = 0.5, a slight partial α-fiber appeared between the G and P components, and it increased till ε = 1.1. As the deformation level increased, this fiber seemed to be “broken” for higher deformation states as indicated by the ODF with φ_2_ = 45°, in which the Brass (B) and Rotated Goss (RtG) texture components were not detected. The S component also seemed to follow the same trend as the α-fiber texture, with a weaker decrease for deformation up to 2.2 in comparison to the B and RtG components. This first features observed during the conventional rolling process, with the α-fiber was found to continuously increased as the deformation increased, as it was reported by Sathiaraj et al. [16] and Haase et al. [18]. The <111> fiber (Cu and A) texture component displayed the strongest increase with the grooved rolling deformation, which firstly confirms the existence of this fiber but may also indicate the presence of twinning deformation mechanism. This deformation by twinning may also occur at lower level of deformation in comparison to conventional rolling. Haase et al. have shown that for conventional rolling, the Cu texture component appeared after a rolling deformation ε = 1.6 [18]. In our study, for a deformation of about 1.1, this component appeared to be the major one in the ODF section and it was found to continuously increase. However, the two components of the <001>RD appeared to have different behavior: as the deformation by groove cold rolling increased, the Cb component tended to progressively disappear, while the G component slightly increased without been affected by the rupture of the α fiber. It is important to note that the G components are both appertaining to <110> along ND and <001> along ND fiber texture, so that their variations may be correlated with the evolution of these two fiber textures.

**Figure 7:**
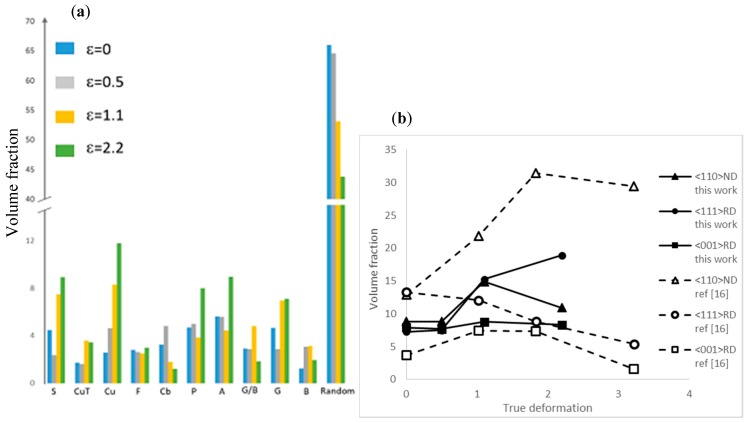
(**a**) Volume fraction of the main principal different detected texture component and (**b**) fiber- texture comparison between groove cold rolling (this work) and conventional cold rolling (ref. [16]).

Figure 7a shows the variation of the volume fraction of each texture component with the increasing deformation by groove cold rolled process, the texture components being obtained from the ODF with a maximum deviation around the ideal orientation of 10° [18]. In addition to the grain elongation as a function to the groove rolling strain (Table 3), a global tendency is a decrease of the random orientation, which could be correlated to the formation of a strong crystallographic texture. It can also be observed that at least 4 texture components reached a volume fraction of 8% after a groove rolling deformation ε = 2.2, which can be directly correlated to the complex texture described above. Figure 7b shows the different volume fraction associated to fiber textures reported previously. The aim here is to compare conventional cold rolling to grooved cold rolling by summing different texture component associated to each fiber texture at different area reduction. The <110>ND component was obtained by summing G, G/B, and B components, <111>RD with Cu and A texture component and <001>RD with Cb and G. One may firstly note that the divergence from the as-annealed state for the two deformation processes may be explained by the different annealing temperatures and by slightly different mechanical post-processing conditions applied to the HEA (see ref. [16]). As expected by the previous analysis, the evolutions of the volume fraction of each fiber texture are clearly different for the two conventional and groove rolling processes. As previously reported by Sathiaraj et al. [16], <110>ND is the main fiber-texture by conventional cold rolling, while the two other studied fibers show a continuous decrease. In the case of groove cold rolling, the <001>RD fiber remained relatively constant from the initial state up to the high deformation state. The two other fibers conserved the same evolution until intermediate deformation state, with a simultaneous increase. For higher deformation level, the rupture of α-fiber shown in Figure 6 (between ε = 1.1 and 2.2) is responsible for the observed decrease of this fiber. In contrast, the <111>RD fiber texture continued to evolve and it became predominant over the other texture components. These final states are closely corresponding to the textures found in FCC materials deformed by swaging or drawing processes, as already published by Otto et al. [15].

To summarize, the texture of CoCrFeMnNi HEA alloy by groove cold rolling may be described as a hybrid texture between the texture obtained by conventional cold rolling and that obtained by swaging or drawing processes. Under high level of deformation, the <111>RD fiber tended to become predominant due to the decrease of the <110>ND fiber. This final state appeared to be very similar to the texture of FCC materials deformed by swaging process.

## 4. Discussion

This study firstly focused on the evolution of mechanical properties of the CrCoFeMnNi HEA during deformation by groove cold rolling. Our results revealed two stages in the evolution of the microhardness, yield strength, and Young’s modulus, with a change of slopes for a groove rolling deformation of about ε = 0.5. Both microhardness and yield strength displayed a strong increase with the strain up to ε = 0.5 by groove cold rolling followed by a moderate increase, to reach values of, respectively, around 400 HV and 1400 MPa for an applied groove rolling deformation ε equal to 2.2. In contrast, the Young’s modulus displayed firstly a decrease of about 15% from its initial value as the groove rolling deformation increased to about ε = 0.5, followed by a slight increase from 175 to 179 GPa as the groove rolling deformation was further increased till ε = 2.2.

The texture analysis revealed that the as-annealed and deformed microstructures presented similar features to those reported in previous studies on CoCrFeMnNi HEA processed by conventional cold rolling. From these considerations, it has been assumed that the deformation mechanisms involved during groove cold rolling were the same as those already reported for CoCrFeMnNi HEA alloy, i.e., dislocation glide and twinning. In order to interpret the variation of the mechanical properties, identification of the main texture components and their evolutions tended to confirm the emergence of a specific crystallographic texture responsible for the change in the microhardness and yield strength as described above. It has been shown that these textures could be interpreted as a hybrid texture between conventional rolling and swaging texture. Also, under high deformation level, our analyses suggested that the swaging type texture tended to become predominant.

The evolution of hardness and yield strength may be regarded through the evolution of the active deformation mechanism. Otto et al. [15], as well as Hasse et al. [18], had reported that plastic deformation was mainly governed by dislocation glide for reduction less than 50% (i.e., ε ~ 2), while both twinning and dislocation glide are active deformation mechanisms beyond this limit for CrCoFeMnNi HEA processed by conventional cold rolling. For alloy processed by groove rolling process, the similar variation in the properties suggested that the deformation mechanisms might also be operating, and that the breakdown occurred for lower value of rolling deformation owing to the biaxial state of stress applied during groove cold rolling. It might also indicate that the work hardening, and the change in the density of dislocation, in CoCrFeMnNi HEA may be more important when deformed under groove cold rolling, i.e., bi-axial compression, than during conventional cold rolling.

The variation of the Young’s modulus followed a different pattern that might be more complicated and less intuitive at this current stage of our investigation. Owing to the strong anisotropy of FCC alloys, such as austenitic Fe and Ni alloys, values of the Young’s modulus are expected to be strongly dependent upon the crystallographic texture [41]. Since the elastic constants of the CoCrFeMnNi HEA have not been reported yet, the Young’s modulus was calculated from the fourth-order elastic compliance tensor S_ijkl_ of the quaternary CoCrFeNi alloy [42]. Without any texture, these elastic compliances give values of 96.1, 232.9, and 442.8 GPa for the Young’s modulus calculated, respectively, along the <001>, <101>, and <111> directions. Taking into account the crystallographic texture expressed by the ODF measured at the different levels of groove rolling deformation, the calculated values of the Young’s modulus have been reported in Table 3. Though the calculated values are larger than those reported in the literature and measured in this study, these results tend to suggest that the Young’s modulus do not change significantly for CoCrFeMnNi HEA with texture corresponding to groove rolling deformation varying between ε = 0 and 1.1. Beyond ε = 1.1, the crystallographic texture oriented toward the orientation <111>, as seen in the IPF (Figure 6) and identified as the <111>RD fiber-texture (Figure 7), induced a marked increase of the Young modulus. Consequently, from this point of view, the crystallographic texture cannot provide a satisfactory explanation for the change in the Young’s modulus detected in our study, which is in agreement with earlier studies [27] undertaken on conventional steels.

As it has been advanced in the introduction, changes in the dislocation arrangement could be responsible for the variation of the elastic properties. Otto et al. [8] explained that the deformation was essentially the results of dislocations gliding at low deformation level and they reported the formation of dislocation cell-structures under higher degrees of deformation. Laplanche et al. [23] have also shown that the dislocation density increased continuously as the deformation increased. Joo et al. [36] have shown that twinning had an effect in term of dynamic Hall-Petch relationship but also led to an accumulation of stacking fault. In addition Benito et al. [27] have demonstrated a strong connection between dislocation structure developed during deformation and decrease in the Young’s modulus. By considering all of these features, and assuming that they are also operating in solid solution deformed by groove cold rolling, it seems reasonable to consider that the change in the Young’s modulus could be mainly governed by the structure of dislocation developed under level of deformation and the gliding of mobile (non-pinned) dislocations within the cells. The stabilized value of the Young’s modulus observed for larger value of the rolled deformation could be explained by modification in the dislocation arrangement resulting from the contribution of the twinning deformation.

## 5. Conclusions

In this study, samples of CoCrFeMnNi HEA were successively deformed by groove cold rolling at different strain levels. The mechanical properties and crystallographic texture were evaluated as a function of the increasing cold rolling deformation, leading to the following conclusions:

- Both the microhardness and the yield strength exhibited similar evolution as the area reduction ratio applied by groove cold rolling increased. A first marked increase until a groove rolling deformation of about 0.5 was followed by a moderate increase under higher strain level. This two-step behavior has been attributed to change in the activated deformation mechanisms, and characterized by a transition from dislocation mechanism and dislocation + twinning deformation mechanism as reported by Otto et al. [8] and Haase et al. [18]. For the groove rolling process, these breakdowns occurred at lower levels of deformation in comparison to conventional cold rolling.
- The Young’s modulus also displayed a two-step variation as the groove cold rolled deformation increased. An approximately 15% decrease from 210 GPa to 175 GPa was observed between annealed HEA samples and those deformed up to about 0.5 by groove rolled process. For higher level of the groove cold rolling deformation, the Young’s modulus value stabilized around 175 GPa. This evolution of the elastic property, which could not be attributed by a modification of the crystallographic texture, has been explained by the transition of operating deformation mechanisms between gliding of non-pinned dislocations within dislocation cell in the first stage, and contribution of twinning for larger values of the groove rolling deformation.
- The texture analysis highlighted a complex crystallographic texture induced by groove cold rolling deformation. This texture has been described as a hybrid texture between the texture developed during conventional rolling and that from swaging or drawing process. This texture was found to consist in a combination of <111> fiber texture along the RD, <110> fiber texture along ND and ND’ and <001> texture along RD. At higher strain level, the <110> fiber texture tended to disappear for the benefit of <111> fiber texture along the RD. This texture was found to be consistent with the bi-axial deformation induced by groove cold rolling but could not account for the decrease in the Young’s modulus.

## Figures and Tables

**Table 1:** Different grooved cold rolling deformation level produced for microhardness and tensile test, grayed background correspond to samples used for electron back scattered diffraction (EBSD) analyses.

*e_i_* (mm)	*e_f_*(mm)	Section Reduction (%)	True Deformation
9.3	9.3	0.0	0.0
-	8.6	14.5	0.2
-	7.6	33.2	0.4
-	7.3	38.4	0.5
-	6.6	49.6	0.7
-	5.4	66.3	1.1
-	4.2	79.6	1.6
-	3.1	88.9	2.2

**Table 2:** Definition of the texture components in the alloy.

Texture Component	Symbol	Euler Angle φ_1_, Φ, φ_2_	Miller Indices
Cb (Cube)	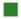	0, 0, 0	{001}<100>
G (Goss)	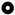	0, 45, 0	{110}<100>
B (Brass)	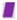	35, 45, 0	{110}<112>
G/B (Goss/Brass)	⨂	74, 90, 45	{110}<115>
Cu (Copper)	<>	90, 35, 45	{112}<111>
A	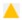	35, 90, 45	{110}<111>
F	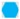	30/90, 55, 45	{111}<112>
P	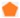	30, 90, 45	{011}<211>
CuT	◆	90, 74, 45	{552}<115>
S	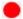	59, 37, 63	{123}<634>

**Table 3:** Values of the length/width aspect ratio of the grains, *r*, and Young’s modulus, *E*, measured along the rolling direction as a function of the groove rolling strain, ε.

ε	0	0.5	1.1	2.2
*r*	1.9 ± 0.7	2.3 ± 0.7	3.4 ± 1.0	5.8 ± 2.0
*E* (GPa)	262.2 ± 5.8	263.0 ± 13.1	262.8 ± 13.1	279.0 ± 12.3
